# Durable response for ampullary and duodenal adenocarcinoma with a nab‐paclitaxel plus gemcitabine ± cisplatin combination

**DOI:** 10.1002/cam4.2181

**Published:** 2019-05-17

**Authors:** Putao Cen, Curtis J. Wray, Songlin Zhang, Nirav C. Thosani, Brian Cuong Dinh, Anneliese Gonzalez, Virginia Mohlere, John Steven Bynon

**Affiliations:** ^1^ University of Texas McGovern Medical School at Houston Houston Texas; ^2^ Memorial Hermann‐Texas Medical Center Houston Texas

**Keywords:** adenocarcinoma, ampullary, cisplatin, CK7, duodenal, gemcitabine, MUC1, nab‐paclitaxel

## Abstract

**Background/Aim:**

There is no standard salvage chemotherapy for metastatic periampullary adenocarcinoma and duodenal adenocarcinoma and the prognosis of those who fail oxaliplatin, irinotecan, and 5FU is dismal. We examined nanoparticle albumin‐bound paclitaxel (nab‐paclitaxel) as salvage therapy for these two malignancies.

**Methods:**

Patients who failed oxaliplatin, irinotecan, and 5FU and whose archival tumors stained immunohistochemical (IHC) tumor positive for CK7 or MUC1 received nab‐paclitaxel and gemcitabine therapy with or without cisplatin.

**Results:**

Three patients, 2 with metastatic ampullary adenocarcinoma and 1 with duodenal adenocarcinoma with positive IHC staining for CK7 or MUC1 who failed 2 lines of chemotherapy with oxaliplatin, irinotecan, and 5FU received nab‐paclitaxel and gemcitabine with or without cisplatin. All achieved excellent tumor response on CT scans with marked falls in tumor markers CA19‐9 and CEA as well as ≥1 year of progression‐free survival. All 3 have continued to survive 2‐3 years since diagnosed with stage 4 metastatic adenocarcinoma.

**Conclusions:**

Nab‐paclitaxel plus gemcitabine with or without cisplatin should be investigated as a standard‐of‐care chemotherapy regimen for patients with ampullary adenocarcinoma and duodenal adenocarcinoma.

## 1 INTRODUCTION

Periampullary carcinomas include ampullary carcinomas and carcinomas of the pancreas, distal bile duct, and periampullary duodenum. Ampullary carcinomas (adenocarcinomas of the ampulla of Vater) arise within the ampullary region distal to the bifurcation of the distal common bile duct and the pancreatic duct. The incidence of ampullary adenocarcinoma has increased over the past 30 years at an annual rate of 0.9%.^1^ Duodenum Adenocarcinoma are considered cancers of the small bowel. Approximately 10 470 new cases of small bowel adenocarcinoma are expected to be diagnosed in the United States in 2018.^2^ The prognosis of patients who relapse or who present with stage 4 metastatic ampullary or duodenal adenocarcinoma is poor.

Due to the rarity of both ampullary and duodenal adenocarcinoma, no large randomized clinical trial has identified a standard chemotherapy regimen for these tumors. Historically, both ampullary and duodenal adenocarcinomas have been treated similarly to cholangiocarcinoma or colon cancer. Standard chemotherapy regimens include gemcitabine plus platinum compounds (cisplatin or oxalipatin), or fluoropyrimidine‐based chemotherapy (FOLFOX or FOLFIRI). These therapies generally achieve a median survival of ≤1 year.^3^, ^4^ In the absence of solid data, neither the National Comprehensive Cancer Network clinical practice guidelines nor the European Society for Medical Oncology provides treatment guidelines or recommendations for management of these malignancies. Thus, novels regimens and innovative approaches for these cancers with poor long‐term outcomes are desperately needed.

## 2 METHODS

We performed immunohistochemical (IHC) tumor staining for CK7, CK20, CDX‐2, MUC1 (Mucin1), and MUC2 on patients' archival tumor specimen for patients with stage 4 ampullary adenocarcinoma and duodenal adenocarcinoma who had failed first‐ and second‐line chemotherapy with oxaliplatin, irinotecan, and 5FU. Diffuse positivity of CK7 or MUC1 was considered as positive. Those patients whose tumors were IHC positive for CK7 or MUC1 were selected to receive nanoparticle albumin‐bound paclitaxel (nab‐paclitaxel) 125 mg/m^2^ and gemcitabine 400‐500 mg/m^2^ with or without cisplatin 25 mg/m^2^, weekly. Tumor response on CT scans with tumor markers, patient progression‐free survival and overall survival were evaluated. A next generation sequencing (NGS) based assay (FoundationOne^®^) were performed on patients' tumors. (Appendix S1) All patients signed informed consent before the study.

## 3 RESULT

In 2016‐2017, we performed IHC on tissues from 3 eligible patients; all 3 patients' tumors stained positive for CK7 or MUC1. Herein, we report 2 cases of ampullary adenocarcinoma and 1 case of duodenal adenocarcinoma with positive IHC tumor staining for CK7 or MUC1 that failed 2 lines of prior chemotherapy with oxaliplatin, irinotecan, and 5FU but successfully achieved durable and exceptional responses to nab‐paclitaxel and gemcitabine with or without cisplatin combinations. The Appendix (online only) provides genomic alterations in patients' tumors under a NGS based assay.

### 3.1 Case 1: Ampullary adenocarcinoma, with peritoneal metastases, bone and soft tissue metastases

**Figure 1:**
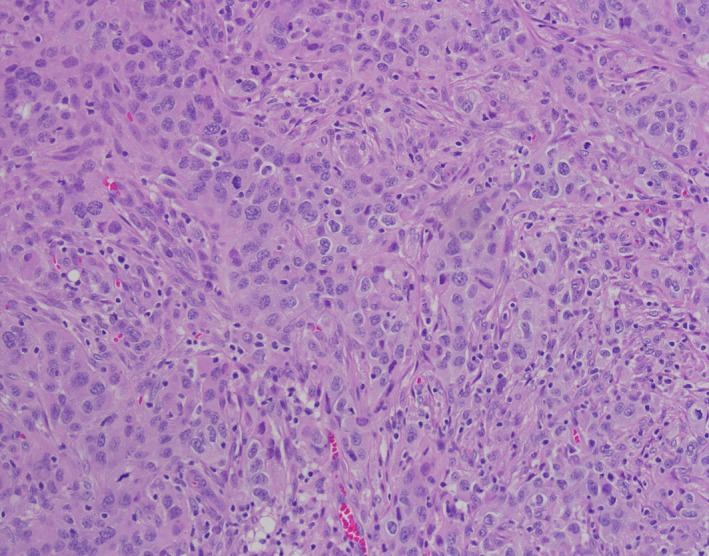
H&E pathology slides showed poorly differentiated adenocarcinoma from excisional biopsy of omental implant

**Figure 2:**
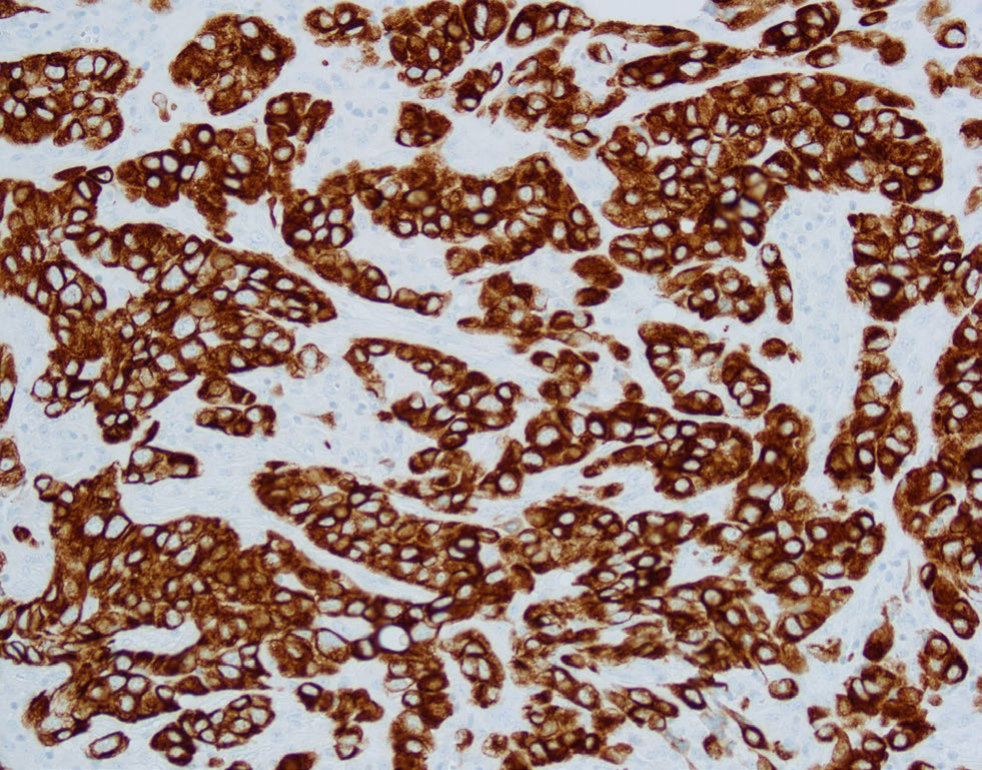
Pathology slides showed metastatic cancer cells are strongly positive for CK7

**Figure 3:**
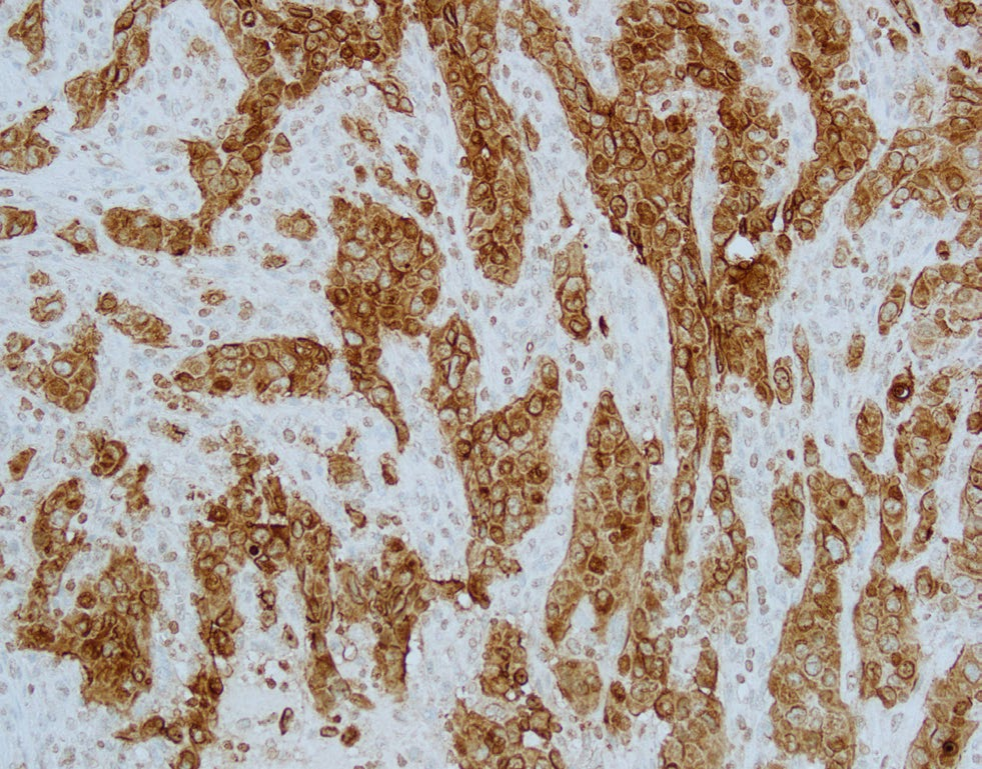
Pathology slides showed metastatic cancer cells are strongly positive for MUC1

**Figure 4:**
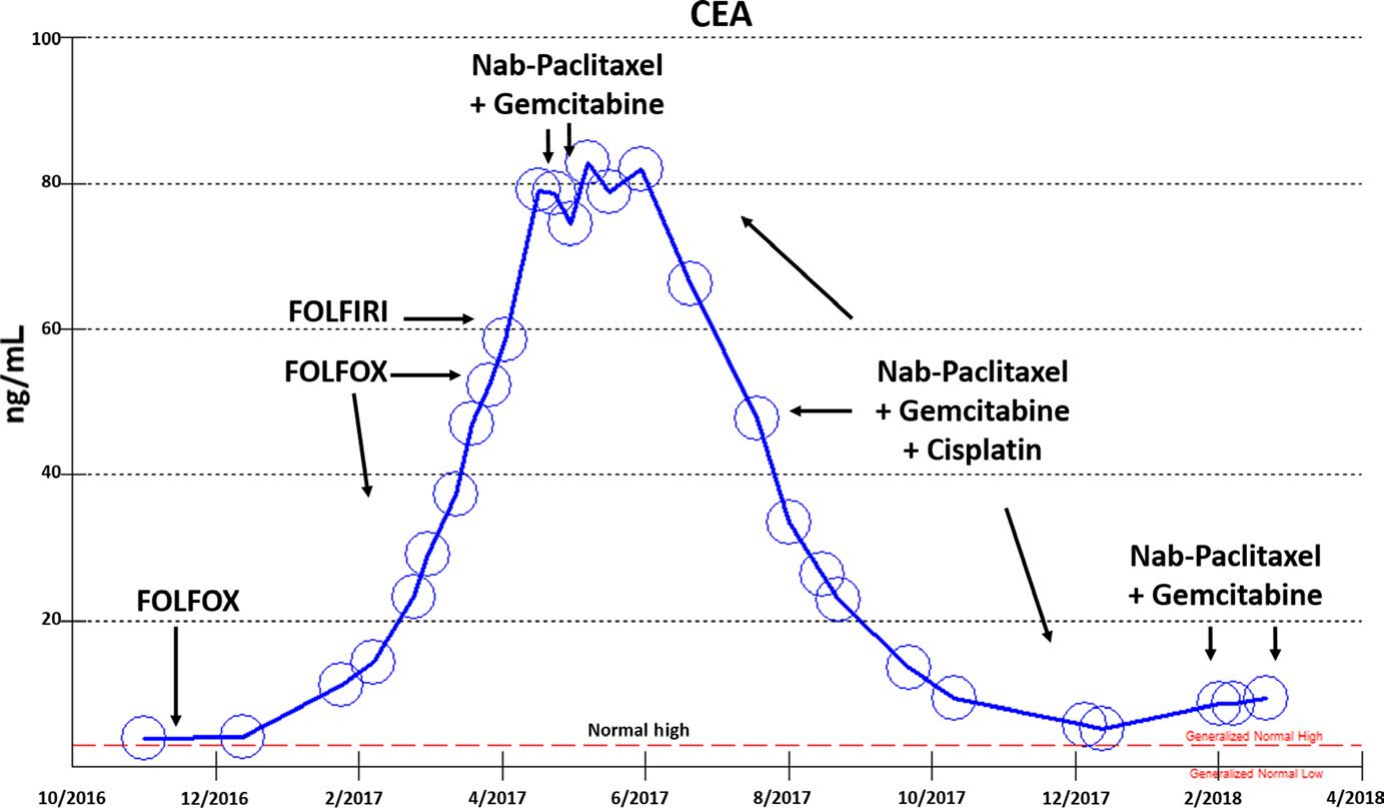
Tumor marker CEA increased during FOLFOX and FOLFIRI chemotherapy, but decreased during gemcitabine nab‐paclitaxel ± cisplatin chemotherapy

A 57‐year‐old white woman presented in early 2016 with sepsis, jaundice, and left upper quadrant pain. An ampullary mass was found and biopsy revealed poorly differentiated adenocarcinoma. At exploratory laparotomy peritoneal metastases were found. Excisional biopsy of a 3‐cm omental mass confirmed poorly differentiated adenocarcinoma. Tumor cells were strongly and diffusely positive for CK7, CK19, MUC1 and negative for CK20, CDX‐2, MUC2 (Figures 1, 2, 3). The patient received FOLFOX for 10 months during which she developed worsening left shoulder and bilateral hips pain. A CT scan showed significant progression of disease in her left shoulder, bilateral hips, and peritoneal metastases, and her CEA level increased to 29 ng/mL. She received 1 dose of FOLFIRI but cancer pain worsened and while CEA levels increased to 37 ng/mL (Figure 4).

**Figure 5:**
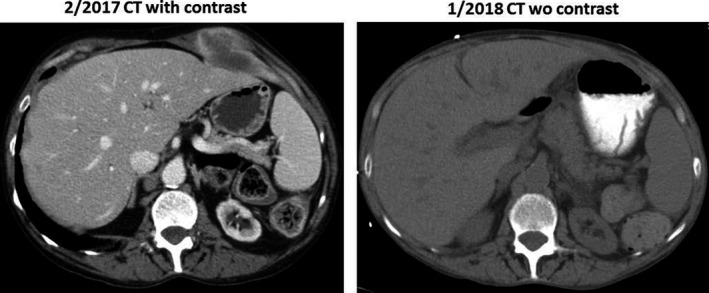
Painful chest wall tumor mass had almost disappeared after new regimen gemcitabine nab‐paclitaxel ± cisplatin chemotherapy

**Figure 6:**
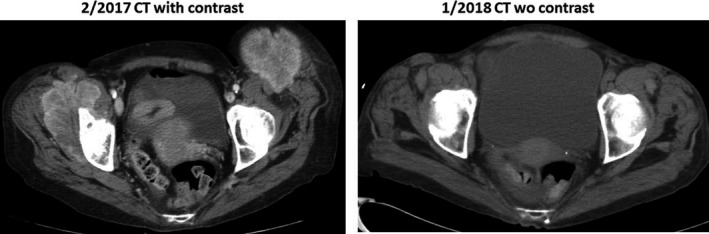
Painful Hip soft tissue tumor mass had almost disappeared after given gemcitabine nab‐paclitaxel ± cisplatin chemotherapy

**Figure 7:**
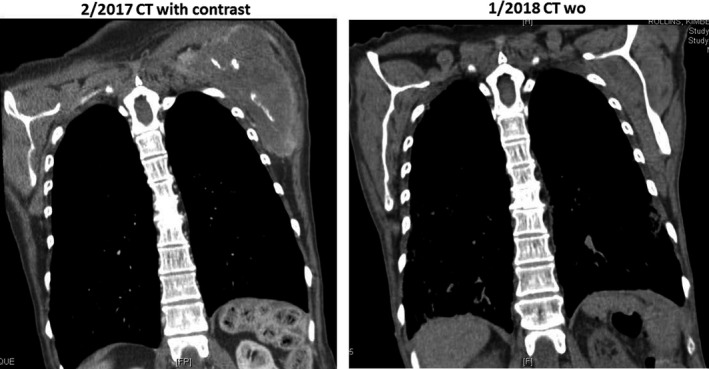
Painful shoulder bony metastasis has significant reduced after given gemcitabine nab‐paclitaxel ± cisplatin chemotherapy

Positive IHC staining for CK7 and MUC1 was consistent with pancreatobiliary‐type ampullary adenocarcinoma. Chemotherapy was decided to switch to gemcitabine 400 mg/m^2^ and nab‐paclitaxel 125 mg/m^2^ weekly. The patient's cancer‐related bony pain rapidly reduced from 10/10 to 1/10 on a pain scale. Because the patient's CEA remained stable during gemcitabine nab‐paclitaxel treatment, cisplatin 25 mg/m^2^ was added to be given weekly, 3 weeks on and 1 week off, for 6 months (Figure 4). The patient reported that the new regimens gave her more energy over time and she gained appetite and weight. Restaging CT scans demonstrated significant tumor reduction compared to prior scans with a fall in tumor marker CEA (Figures 4, 5, 6, 7). The gemcitabine and nab‐paclitaxel regimen has been continued, with an ongoing tumor response for >1 year (3/2017‐5/2018). Cisplatin was placed on hold due to increased creatinine.

### 3.2 Case 2: Stage IV ampullary adenocarcinoma with bulky mediastinal lymph node metastasis

**Figure 8:**
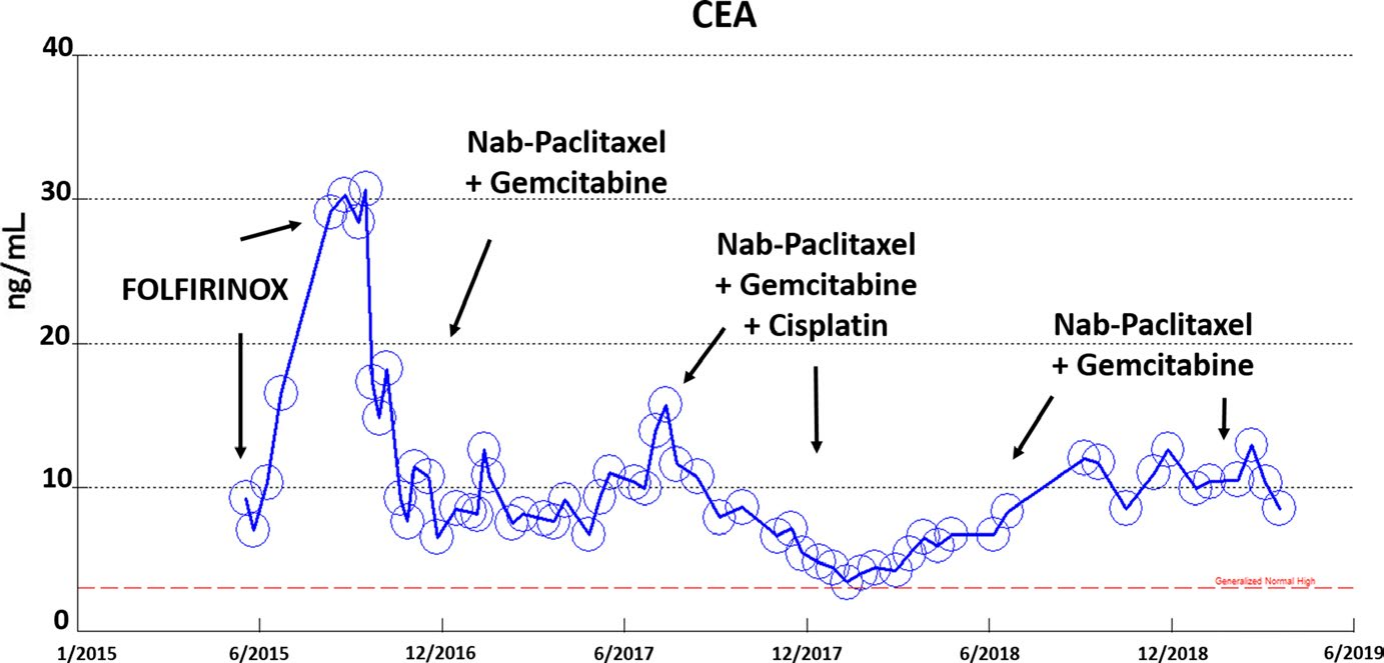
Tumor marker CEA increased during FOLFIRINOX chemotherapy, but decreased during gemcitabine nab‐paclitaxel ± cisplatin chemotherapy

**Figure 9:**
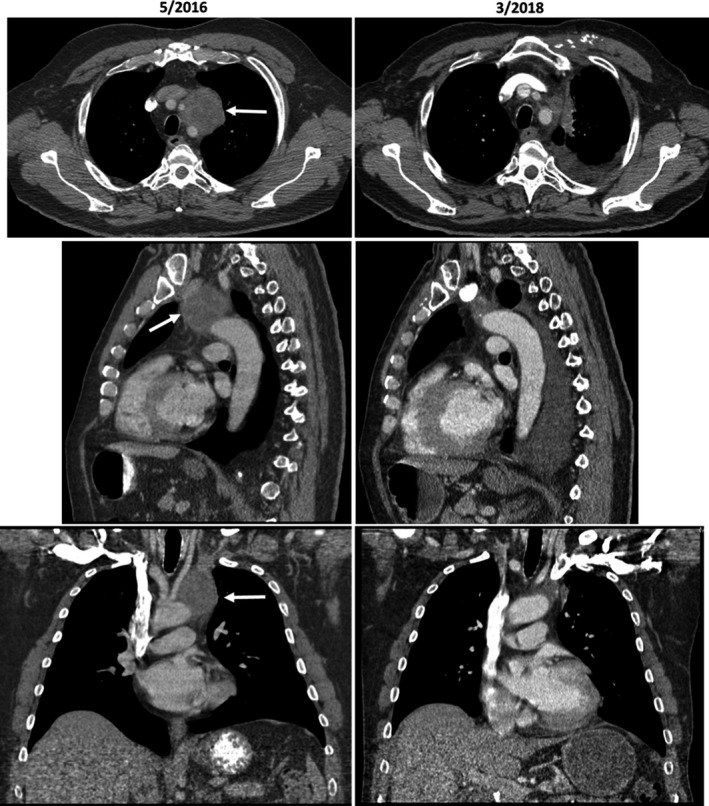
Large mediastinal nodal metastasis (causing have left vocal cord paralysis) had significant reduced after given gemcitabine nab‐paclitaxel ± cisplatin chemotherapy

A 60‐year‐old white man presented with jaundice (total bilirubin of 12 mg/dL) in late 2015. A 2‐cm ampullary mass involved the distal common bile duct was found and biopsy showed a poorly differentiated adenocarcinoma involving the small‐intestine mucosa. Initial CT scans showed biliary duct dilatation, multiple 1 cm reginal lymph node enlargement and a large 4‐cm mediastinal lymph node. Biopsy of the large mediastinal showed poorly differentiated adenocarcinoma that stained positive for CK7 but negative for CDX2, TTF‐1, NapsinA, and CK 20, consistent with an ampullary origin. The patient received FOLFOX for 5 months at an outside institute during which the patient noticed progressive voice hoarseness and was discovered to have left vocal cord paralysis. In May 2016, restaging CT scans shows the mediastinal mass had increased to 5 cm (Figure 9). Due to disease progression, chemotherapy was switched to FOLFIRINOX for 2 months. Concurrent conventionally fractionated radiotherapy with 60 Gy in 30 fractions was also aimed to the patient's bulky mediastinal node. In September 2016, after concurrent chemoradiation, chest CT showed the mediastinal node had slightly decreased in size but several metastatic nodular pulmonary lesions had appeared with an increase in CEA tumor marker to 35 ng/dL, confirming continued tumor progression (Figure 8).

Because the patient's tumor IHC profile (CK7 positivity) was consistent with pancreatobiliary‐type ampullary adenocarcinoma, therapy was switched to gemcitabine 400 mg/m^2^ and nab‐paclitaxel 125 mg/m^2^, given once every 10 days. On this regimen, the CEA levels rapidly decreased with disappearance of metastatic lung lesions and improvement in hoarseness. Because the patient's CEA decline reached a plateau after 10 months of gemcitabine and nab‐paclitaxel regimen, in July 2017, cisplatin 25 mg/m^2^ was added to the regimen, given 2 weeks on and 1 week off, for 3 months. Subsequent restaging with CT and EUS showed a marked decrease in mediastinal lymph node size to 1.4 cm and further CEA decrease to 4.8 ng/dL (Figures 8 and 9). EUS RFA in December 2017 was used to ablate the 1.4‐cm mediastinal node. As of this report, the patient is on maintenance chemotherapy with gemcitabine 300 mg/m^2^ and nab‐paclitaxel 125 mg/m^2^ weekly, 2 weeks on and 1 week off, and has maintained a stable, ongoing response for close to 3 years (9/2016‐present). Cisplatin is on hold due to increased creatinine and eGFR of 40 mL/min/1.73 m^2^.

### 3.3 Case 3: Duodenal adenocarcinoma with peritoneal and liver metastases

A 52‐year‐old woman presented in April 2014 with jaundice, pruritus, nausea, and vomiting. A duodenal mass was found obstructing her biliary tree. She received a Whipple procedure. Surgical pathology showed a 6.5‐cm adenocarcinoma, moderately differentiated with partial mucinous differentiation, arising in small intestinal tubulovillous adenoma with high‐grade dysplasia, invasive into peri‐intestinal soft tissue, with contiguous extension into pancreas, and 7 of 25 lymph nodes were involved with metastatic carcinoma. The patient's disease was pathological stage T4N2M0. IHC staining was positive for CK7, CK20, CDX‐2, and MUC‐1 (negative staining for MUC‐2), employing a cutoff threshold for positivity of 25%. Subsequently, the patient received 6 cycles of FOLFOX adjuvant chemotherapy.

**Figure 10:**
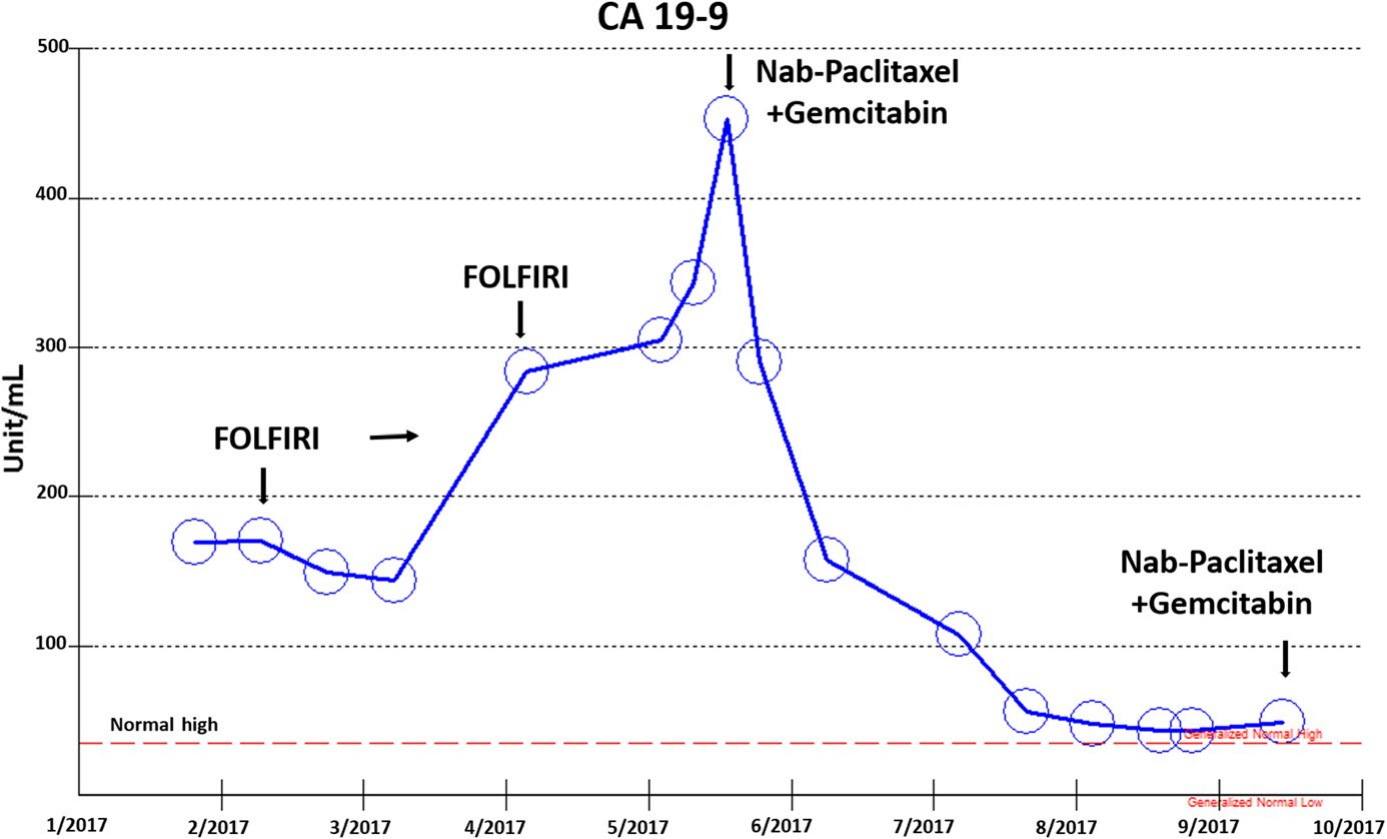
Tumor marker CA19‐9 increased during FOLFIRI chemotherapy, but decreased during gemcitabine nab‐paclitaxel chemotherapy

Two years after her initial Whipple surgery, surveillance CT revealed development of extensive peritoneal metastatic disease in the abdomen and new hepatic hypo‐densities consistent with tumor recurrence. After 10 months of palliative FOLFIRI chemotherapy starting in June 2016, her cancer progressed on both CT scans and tumor marker CA19‐9. Because her tumor's immunophenotypic profile was positive not only for MUC1 and CK7 but also for CK20 and CDX‐2, her tumor was considered ambiguous with both pancreaticobiliary‐type and intestinal‐type features. Nab‐paclitaxel 125 mg/m^2^ plus gemcitabine 300‐400 mg/m^2^ was chosen as third‐line salvage chemotherapy with each given over 30 minutes weekly, 3 weeks on and 1 week off. Tumor response was demonstrated by CT scans and tumor marker CA19‐9 markedly declined from 452unit/ml to 42unit/ml and has remained stable for 1 year (5/2017‐3/2018) (Figure 10).

## 4 DISCUSSION

The current recommended chemotherapy regimens for ampullary and duodenal adenocarcinoma were extrapolated from limited small phase II trials, and the trials mostly for cholangiocarcinoma.^3^, ^4^, ^5^ The largest and most recent phase III randomized multicenter ABC‐02 trial included patients with both locally advanced (25%) and metastatic bile duct (n = 242), gallbladder (n = 148), or ampullary (n = 20) cancer. The median overall survival was 11.7 months with first line gemcitabine plus cisplatin combination treatment, compared with 8.1 months for gemcitabine alone.^3^ A recent small single‐center phase II studied 30 patients, including 23 (77%) with small bowel cancer (18 of duodenal origin and 5 of jejunal/ileal origin) and 7 patients (23%) with ampullary adenocarcinoma (5 of pancreaticobiliary subtype, 1 of mixed subtype, and 1 of intestinal subtype) who were given first line capecitabine and oxaliplatin plus bevacizumab and had median overall survival of 12.9 months.^5^

Ampullary adenocarcinoma's histological subtypes and IHC staining patterns have been shown to have prognostic significance. However, conflicting data have been reported about the frequency of the two major subtypes of ampullary cancers (intestinal and pancreatobiliary type) due to the absence of reliable histomorphological standard or IHC markers for differential diagnosis. Survival in patient with tumors of pancreatobiliary type or CK7/MUC1‐positive or CDX2‐negative ampullary tumors seem to have a worse prognosis which is similar to that of patients with pancreatic cancer.^6^, ^7^, ^8^ In a Korean study of 37 patients with ampullary adenocarcinoma who underwent Whipple procedure, half of them (18/37) had CK7‐positive tumors. Multivariate analysis showed that CK7+/CK20− was a significant independent factor predicting poorer survival, whereas nodal positivity status was not predictive.^7^ In a retrospective cohort of 72 patients from Australia who underwent surgical resection for adenocarcinoma of the ampulla of Vater, 18 (25%) patients with a histo‐molecular pancreaticobiliary phenotype (MUC1‐positive, CDX‐negative) had significantly worse outcomes than those with an intestinal phenotype (CDX‐positive, MUC1‐negative), with a median survival of 16 versus 116 months.^8^ In an Italian study of 53 resected ampullary cancer cases, MUC1 expressed in 75% (40/53) of the total cases, with 97% (29/30) positive among pancreatobiliary type and 47% (11/23) positive among intestinal type cancers; CDX2 expressed in 60% (32/53) of the total cases, with 30% (9/30) positive among pancreatobiliary type and 100% (23/23) positive among intestinal type cancer. A longer survival was correlated with the expression of CDX2, when using a nuclear labeling cutoff of >10% cells (*P* = 0.14).^9^

Nab‐paclitaxel is nanoparticle albumin‐bound paclitaxel. In 2013, the FDA approved nab‐paclitaxel for the first‐line treatment of patients with metastatic adenocarcinoma of the pancreas. This indication was based on the multinational MPACT trial of 861 patients who were randomized to receive either the combination of nab‐paclitaxel (125 mg/m^2^) plus gemcitibine (1000 mg/m^2^, each given weekly, on days 1, 8, and 15 every 28 days) or gemcitabine alone (1000 mg/m^2^ weekly for 7 weeks, then on days 1, 8, and 15 every 4 weeks). Adding nab‐paclitaxel to gemcitabine was associated with a significantly higher objective response rate (23% vs 7%) and a significantly longer median overall survival (8.5 vs 6.7 months).^10^

Kapp et al^11^ from Germany retrospectively reported an exceptional response to nab‐paclitaxel and gemcitabine in 1 patient with refractory ampullary adenocarcinoma whose tumor IHC staining pattern was CK7‐positive and CK20‐ and CDX2‐negative.

In our study, we identified a distinct clinically relevant (pancreaticobiliary) phenotype by refining histologic classification with IHC staining (CK7 or MUC1 positivity) criteria and studied these patients for our novel chemotherapy regimen.

All 3 of our patients in the study had failed 2 lines of chemotherapy Oxaliplatin, Irinotecan, and 5FU with the diagnosis of stage 4 metastatic adenocarcinoma. By this criteria, their survival was expected to be dismal, with weeks or a few months of life expectancy, without any effective salvage chemotherapy. Both regimens—nab‐paclitaxel plus gemcitibine and nab‐paclitaxel plus gemcitibine with cisplatin—were very well‐tolerated for long‐term use in our patients, partially because gemcitabine was given at less than half (300‐500 mg/m^2^) of the standard dose (1000 mg/m^2^). Despite this, the efficacy of the regimens were not compromised and may actually have been improved. This was possibly due to the reduction in gemcitabine dosage–related grade 3‐4 cytopenic toxicity. Thus, our patients were better able to keep their weekly chemotherapy administration on schedule, and, in turn, achieved better tumor response and prolonged survival.

Compared to other literatures, our study shows longer survival of 2‐3 years and marked tumor reduction that were successfully achieved in chemo‐resistant stage 4 metastatic ampullary and duodenal adenocarcinoma by using our novel approach in third line salvage chemotherapy with nab‐paclitaxel plus gemcitabine ± cisplatin.

One limitation of our study is that we have not administered nab‐paclitaxel to patients whose ampullary and duodenum adenocarcinomas are negative for both CK7 and MUC1. We plan to continue recruiting more patients and increase our sample size. If those with negative markers also achieve good response, it would suggest an even broader indication of nab‐paclitaxel for all ampullary and duodenum adenocarcinomas.

Nevertheless, nab‐paclitaxel plus gemcitibine with or without cisplatin should be considered as a standard‐of‐care chemotherapy regimen for patients with ampullary and duodenal adenocarcinoma. Further studies are urgently needed in these two rare cancers.
