# Supplementary material for: Durable response for ampullary and duodenal adenocarcinoma with a nab‐paclitaxel plus gemcitabine ± cisplatin combination
Source: Cancer Med. 2019 May 17;8(7):3464–70. doi: 10.1002/cam4.2181 (PMC6601707; doi:10.1002/cam4.2181)
Supplement: Supplementary file 1 [file CAM4-8-3464-s001.docx]

Supplement

Appendix

**Case 2: Stage IV Ampullary adenocarcinoma with bulky mediastinum lymph node metastasis**

[Appendix] Case 2. mediastinum lymph node tumor was identified with the following genetic alterations under a next generation sequencing (NGS) based assay (FoundationOne®): ATM (K2811fs*46, splice site 5497-1G>T), ERBB2 (R678Q), KRAS (G12D), PALB2 (G937*), TSC2 (S1431L), CDK12 (G736*), RNF43 (R132*), ARID1A (L2281fs*104), ARID2 (splice site 1498+2T>C), CDC73 (splice site 423+1G>A), CDKN2A (p16INK4a R58 and p14ARF P72L), CREBBP (G1335, Q1968, splice site 85+2T>C), CSF1R (splice site 2555-2A>G), DNMT3A (K140fs*22), EP300 (splice site 4453-1G>A), HGF (P1875), KEL (R516), MLL2 P3466fs*2, splice site 4584-1G>T), MSH2 (G692E), MSH6 (G674, S1188fs*6, Y994), PTPN11 (G503V), RAD50 (R656), RANBP2 (E844), RB1 (I848fs*7, R73), RUNX1 (R201Q), SLIT2 (P926L), SPEN (R1371), TET2 (G773), TPS3 (E294, R175H), and Microsatellite status (MS) – Stable.

**Case 3: duodenal adenocarcinoma with peritoneal and liver metastases**

[Appendix] Case 3. A Next-generation sequencing (NGS) based assay (FoundationOne®) had identified following genomic alteration in her original duodenal tumor: ERBB3(G284R), KRAS (G12A), PIK3CA (H450_P458del), CTNNB1 (S45F), MLL2 (P1460fs*46), PIK3R1 (T576del), SMAD4 (G386D, R135*), microsatellite status Stable, and Tumor mutation burden (TMB) as intermediate – 6 mutations/MB.
